# Characterization of the Methylthioadenosine Phosphorylase Polymorphism rs7023954 - Incidence and Effects on Enzymatic Function in Malignant Melanoma

**DOI:** 10.1371/journal.pone.0160348

**Published:** 2016-08-01

**Authors:** Katharina Limm, Katja Dettmer, Jörg Reinders, Peter J. Oefner, Anja-Katrin Bosserhoff

**Affiliations:** 1 Institute of Biochemistry, Friedrich-Alexander University of Erlangen-Nürnberg, Erlangen, Germany; 2 Institute of Functional Genomics, University of Regensburg, Regensburg, Germany; 3 Comprehensive Cancer Center Erlangen, CCC Erlangen-EMN, Friedrich-Alexander University of Erlangen-Nürnberg, Erlangen, Germany; University of Alabama at Birmingham, UNITED STATES

## Abstract

Deficiency of methylthioadenosine phosphorylase (*MTAP*) supports melanoma development and progression through accumulation of its substrate 5’-methylthioadenosine (MTA), which leads amongst others to a constitutive inhibition of protein arginine methyltransferases (PRMTs) and activation of the transcription factor AP-1 via the receptor ADORA2B. Genetic association studies have also suggested that genetic polymorphism in *MTAP* may modulate the risk of melanoma. Here, we investigated the only globally common non-synonymous single nucleotide polymorphism (SNP) reported to date for *MTAP*. The SNP rs7023954 is located in exon 3 (c.166G>A), and leads to the conservative substitution of one branched-chain amino acid residue (valine) for another (isoleucine) at position 56 (p.Val56Ile). Whereas genotype frequencies in normal and primary melanoma tissues or cell lines were in Hardy-Weinberg equilibrium based on cDNA amplicon sequencing, a marked (P = 0.00019) deviation was observed in metastatic melanoma tissues and cell lines due to a deficit of heterozygotes. Enzyme assays conducted on the co-dominantly expressed alleles revealed no difference in the conversion rate of MTA to adenine and 5-methylthioribose-1-phosphate, indicating that this known enzymatic activity does not modulate the tumor suppressive function of MTAP.

## Introduction

The incidence of malignant melanoma is steadily increasing [1]. Melanoma is a highly invasive cancer characterized by early metastasis and rapid development of resistance to current therapeutic approaches [2]. In the past years, great progress has been made in our understanding of the molecular pathobiology of melanoma. In particular, the identification of driver mutations in genes encoding proteins that play a role in the MAPK pathway, such as BRAF, NRAS, and MEK (MAPK kinase), has spurred the development of novel targeted treatment strategies [3,4]. Another gene that has received increasing interest is methylthioadenosine phosphorylase (*MTAP*). It catalyzes the first step in the methionine salvage pathway by phosphorylating S-methyl-5'-thioadenosine (MTA), a major byproduct of the polyamine metabolism, to adenine and 5-methylthioribose-1-phosphate [5,6]. Many tumors lack expression of *MTAP*, due either to its loss at the 9p21 locus or to hypermethylation of its promoter region [5–8]. *MTAP* deficiency results in the accumulation of MTA [9,10]. High intracellular levels of MTA have been shown to cause deregulation of protein arginine methyltransferases (PRMTs) [8,11]. Further, extracellular MTA affects cellular signaling [8,12] and proliferation [13], not only of cancer cells but also of stromal cells including lymphocytes and fibroblasts, via induction of melanoma-relevant genes such as the growth factors VEGF and bFGF and the metalloproteinases MMP9 and MMP14 [7]. This MTA-based effect could not be observed in melanocytes. Further, it could be shown, that MTA treatment leads to an activation of the transcription factor AP-1 [7], which is highly active in melanoma and influences the expression of a variety of regulators of cell mechanisms involved in melanoma development and metastasis [14]. Activation of AP-1 is modulated by the binding of MTA to the adenosine receptor A2B [12].

In recent years, numerous single nucleotide polymorphisms (SNPs) have been reported for the *MTAP* locus on chromosome 9p21 to dbSNP, including 120 missense polymorphisms, all but one of which are rare or show high population specificity. The only exception is rs7023954, which is located in exon 3 (c.166G>A) of *MTAP* and leads to the exchange of valine for isoleucine (p.Val56Ile) [6]. It is found worldwide with a minor allele frequency of 0.3882 and an average observed heterozygosity of 0.475. Spurred by reports of an association of the *MTAP* locus with melanoma risk [15,16], this study aimed at investigating the frequency of rs7023954 genotypes in normal skin as well as primary and metastatic melanoma tissues and cell lines. Further, we studied allele-specific expression of MTAP at the protein level in trypsin-digested whole-cell extracts using liquid chromatography-mass spectrometry and stable isotope-labeled proteotypic peptides specific for either allele. Finally, we measured enzyme activity of the alleles both in a rabbit reticulocyte lysate translation system as well as in transiently and stably transfected cells.

## Material and Methods

### Cell lines, culture conditions and tissue samples

Melanoma cell lines Mel Juso, Mel Ho, Mel Ei, Mel Wei, 501Mel, SkMel3, SkMel28, Mel Im, Mel Ju, A375, HMB2, and NHEMs were described in detail previously [6,8,17]. Briefly, cell lines Mel Juso (DSMZ: ACC74), Mel Ho (ATTC: ACC62), Mel Wei, and Mel Ei were derived from primary cutaneous melanomas; Mel Im, Mel Ju, 501Mel, A375 (ATTC: CRL-1619), SkMel3 (ATTC: HTB-69), SkMel28 (ATTC: HTB-72), and HMB2 were derived from metastases of malignant melanomas. Melanoma cell lines WM35, WM793, WM1366, WM3211 (primary cutaneous melanomas) and WM9 and WM293A (metastases of malignant melanoma) were a kind gift by Dr. M. Herlyn (Wistar Institute, Philadelphia, PA, USA) and cultivated as described in Tu2% medium [18]. NHEMs were derived from neonatal skin. Isolation and cultivation of NHEMs were described previously [19]. Generation of stably transfected Mel Juso clones (Mock N, KSNV-01 and KWT-04) was achieved by culturing the cells one day after transfection in selection medium, containing 0.6 mg/mL G418 (Sigma-Aldrich, Taufkirchen, Germany). After 14 days of selection, individual G418-resistant colonies were sub-cloned. Expression of *MTAP* in these clones was determined by qRT-PCR and Western Blot. NHDF cells were obtained from Promocell (Heidelberg, Germany). Primary human dermal fibroblasts (NHDF1-6) were cultured as described previously [20]. Cells were maintained in DMEM or RPMI1640 (Sigma-Aldrich, Deisenhofen, Germany) supplemented with penicillin (400 U/mL), streptomycin (50 μg/mL), and 10% fetal bovine serum (FBS) (all from Sigma-Aldrich). The cell lines Mel Juso, Mel Ei, Mel Ho, Mel Wei, Mel Im, HMB2, A375, Mel Ju, 501Mel, SKMel3 and SKMel28 were incubated in a humidified atmosphere containing 8% CO_2_ at 37°C. Cell lines from the Wistar Institute were cultured at 5% CO_2_ and 37°C. Cells were split at a 1:3 to 1:5 ratio every 4 days.

### Tissue specimens

Tissue specimens of snap-frozen melanoma primary tumors (PT60, PT62, PT71, PT87, PT93, PT97, PT104, and TB148) and melanoma metastases (TB43, TB50, TB80, TB90, TB91, TB95, TB101, TB135 and TB147) with clear-cut pathological classification were obtained from the tissue collection of the Institute of Pathology (University of Regensburg, Germany). Sampling and handling of patient material were carried out in accordance with the ethical principles of the Declaration of Helsinki. Use of human tissue material (NL3, NH14, NL146 TB88, TB89, NH118, NH119, NH120, TB121, TB122; TB60, TB62, TB71 TB87, TB93, TB97, TB104, TB148; TB43, TB50, TB80, TB90, TB91, TB95, TB101, TB135, and TB147 obtained from the Department of Dermatology, University Hospital of Regensburg, Germany) had been approved by the local ethics committee of the University of Regensburg (application number 09/11 and 03/151).

### RNA isolation and reverse transcription

Total cellular RNA was isolated from cultured cells using the E.Z.N.A^®^ Total RNA Kit I (Omega Bio-Tek/ VWR, Darmstadt, Germany) according to the manufacturer’s instructions. cDNAs were generated from 500 ng total RNA using SuperScript II Reverse Transcriptase Kit (Invitrogen, Groningen, Netherlands) as described previously [21].

### Amplification and sequencing of *MTAP*-Exon 3

For PCR amplification of exon 3 of *MTAP*, 1 μL cDNA (extracted from tissue or cell lines, or 2 μL genomic DNA (extracted from tissue or cell lines) were added to 0.5 μL of forward and reverse primers (MTAP-for89: 5’-GCCCACTGCAGATTCCTTTC-3’; MTAP-rev416: 5’-GGTCTCATAGTGGTCCTGTC-3’; each 20 μM), 5 μL PCR reaction buffer (10x), 0.5 μL dNTP mix (each 10 mM), and 0.5 μL (2.5 Units) FastStart Taq DNA Polymerase dNTPack (Roche, Mannheim, Germany) in a total reaction volume of 50 μL. The following PCR program was used: 5 min at 95°C (initial denaturation); 60 s at 95°C (denaturation); 30 s at 61°C (annealing), 30 s at 72°C (elongation), repeated 35 times. PCR products were evaluated by gel electrophoresis using a 1.5% agarose gel. Before sequencing, amplicons were purified by precipitation with 30% PEG-8000 (Sigma-Aldrich) to remove unincorporated primers and dNTPs. Purified PCR products were given to GeneArt AG/ Thermo Fisher Scientific Inc. (Regensburg, Germany) as complete mix of 10 ng per 100 bp of PCR-product-length and 10 μM Primer MTAP-Ex3for 5’-CATGCATGCAGCCATCTG-3’ or MTAP-Ex3rev 5’- CGTCAGACCTGTATCTTTAC-3’ for Sanger sequencing.

### Analysis of gene expression by qRT-PCR

Quantitative real time-PCR analysis of *MTAP* was performed using specific primers (MTAPfor2: 5’-GCGAACATCTGGGCTTTG-3’; MTAPrev2: 5’-GCACCGGAGTCCTAGCTTC-3’) and a LightCycler^®^ 480 (Roche, Mannheim, Germany) as described previously [22]. ß-Actin (ß-Actin for: 5′-CTACGTGGCCCTGGACTTCGAGC-3′; ß-Actin rev: 5’-GATGGAGCCGCCGATCCACACGG-3′) was used as a housekeeping gene for normalization. The annealing and melting temperatures were optimized for each primer set. All experiments were repeated at least three times.

### Transfection vector

The coding sequence of *MTAP* was cloned into pCMX-PL1. By specific PCR-reaction using Primer MTAP89for and MTAP987rev the coding sequence of MTAP was amplified [6]. Using the TA Cloning^®^ Kit (Invitrogen, Groningen, Netherlands), the PCR product was cloned into the TOPO-Cloning Vector according to the manufacturer’s instructions. Full-length (CDS) *MTAP*-cDNA was then cloned into the pCMX-PL1 via KpnI/EcoRI (NEB, Frankfurt, Germany) generating the expression constructs MTAP-56V and MTAP-56I. The respective clones were confirmed by sequencing using MTAPfor89 primer.

### Transfection experiments

For re-expression of *MTAP*, 2x10^5^ Mel Juso cells were seeded into 6-well plates and transfected with 0.5 μg of the expression construct (MTAP-56V or MTAP-56I) or pCMX-PL1 vector using Lipofectamine^®^ LTX reagent (Life Technologies, Darmstadt, Germany) according to the manufacturer’s instructions. Twenty-four hours after transfection the medium was changed and the cells were incubated for another 24 h, before supernatant was collected and stored at -80°C until metabolite analysis. Transfected cells were split for mRNA and protein isolation, as well as for metabolite analysis (1x10^6^ cells). All transfections were repeated at least three times.

### *In vitro* translation in a rabbit reticulocyte lysate translation system

Using the TNT^®^Quick Coupled Transcription/Translation System (IVT; Promega, Madison, WI, USA) according to the manufacturer’s instructions, pCMX-PL1, MTAP-56V and MTAP-56I were used for recombinant protein expression. The master mix containing all supplements and 1 μg of either vector was incubated at 30°C for 90 min. The translation efficiency of each sample was determined by Western Blot analysis. The recombinant proteins were stored at -20°C until use.

### Western Blot

For Western Blot analysis, 5 μL of *in vitro* translation mix or 40 μg of RIPA-cell lysates were loaded per lane, and performed as described previously using anti-MTAP antibody (0.5 μg/mL ab55517, Abcam, Cambridge, UK) and anti-ß-actin (1:5000; Sigma-Aldrich) [8]. All Western Blots were repeated three times. Protein bands in western blots were quantified using computer based colorimetric quantification analysis (freeware: ImageJ v1.33 downloaded from the NIH website (http://rsb.info.nih.gov/ij)).

### Enzyme kinetics

For analyzing MTAP-enzyme-kinetic, the *in vitro* translation mix with the recombinant proteins or the control, respectively, was diluted ¼ in PBS. Subsequently, 1 μL of the dilution was added to MTA (10 μM) in 20 μL PBS and incubated at 37°C for defined periods of time (30 s to 30 min). The reaction was stopped by adding 600 μL of 80% MeOH, snap-frozen and stored at -80°C until measurement. All samples were prepared in triplicates and the MTAP-enzyme-kinetic was repeated three times.

### MTA extraction and analysis by liquid chromatography-electrospray ionization-tandem mass spectrometry (LC-ESI-MS/MS)

For analysis of MTA in cell culture medium, cells were cultured for 24 h. Subsequently, supernatant was collected, centrifuged (1200 rpm), snap-frozen, and stored at -80°C. For determination of intracellular MTA, cells were washed with PBS and harvested by incubation in a solution containing 0.05% (w/v) trypsin and 0.02% (w/v) EDTA. Trypsinization was stopped after 2 min with cell culture medium. After centrifugation (1200 rpm, 4 min), the supernatant was removed, the cell pellet was washed with PBS buffer and the cell number was determined by counting the trypsinized cells. The cell pellet was snap-frozen and stored at -80°C. Samples were further processed as described previously [9].

### Analysis of MTAP-56V and MTAP-56I by MS

Cell pellets were lysed by sonification in 300 μL of 100 mM ammonium bicarbonate buffer (pH 7.8) for 10 min. After centrifugation protein concentrations were determined via UV absorption at 280 nm using a NanoDrop2000 system (NanoDrop Instruments, Wilmington, DE, USA). Fifty μg of protein per sample was used for digestion using the RapidACN protocol [23]. Two μg of the resulting peptide mixtures were supplemented with 100 fmol each of the stable isotope-labeled peptides NVDCVLLAR(+10) and NVDCILLAR(+10) and subjected to MRM-HR analysis on a TripleTOF5600+ mass spectrometer (SCIEX GmbH, Darmstadt, Germany) coupled to a Ultimate3000 nano-HPLC (Dionex GmbH, Idstein, Germany). A 28-min-gradient from 4%-40% B (A: 0.1% formic acid; B: 0.1% formic acid in acetonitrile) was used for separation on a 75 μm I.D., 25 cm long C18-column (Acclaim PepMap, 3 μm, 100 Ǻ, flow rate 300 μL/min) with precolumn concentration (Acclaim PepMap, 100 μm I.D., 2 cm, 5 μm, 100 Ǻ, flow rate 5 μL/min). MS/MS-spectra were acquired for 0.5 s each and quantification was accomplished using the Skyline software (MacLean B, et al., Skyline: an open source document editor for creating and analyzing targeted proteomics experiments, Bioinformatics, 2010) based on the transitions from the doubly charged precursors to the singly charged fragment ions y_5_, y_6_ and y_7_.

### Statistical analysis

Results are expressed as either mean ± S.E.M. (range) or percent. Comparison between groups was performed using one-way ANOVA with Bonferroni post-hoc test. A p-value <0.05 was considered statistically significant (ns: not significant). All calculations were performed using GraphPad Prism software (GraphPad Software Inc., San Diego, CA, USA). Testing of rs7023954 genotypes for Hardy-Weinberg equilibrium (HWE) was performed at https://www.cog-genomics.org/software/stats using Fisher’s exact test and Lancaster’s mid-P correction for better control over type I error rates [24]. Both genotypic and allelic odds ratios were calculated based on genotype and allele counts, respectively, for rs7023954 in control and primary melanoma samples [25]. The null hypothesis of no association with disease was tested by means of a χ^2^ test for independence of the rows and columns of 2x2 and 2x3 contingency tables.

## Results

### Genotyping of *MTAP* rs7023954

**Fig 1:**
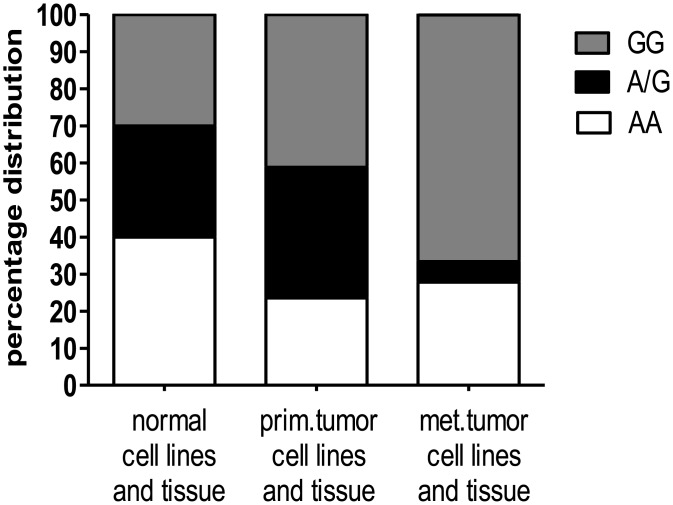
Determination of *MTAP* rs7023954 in cell lines and tissue samples. The non-synonymous *MTAP* SNP c.G166A was genotyped by sequencing of cDNA that had been produced from total RNA extracted from both normal as well as primary (prim.) and metastatic (met.) melanoma cell lines and tissues. Distribution of the genotypes AA, AG, and GG is shown. A detailed description of the genotypes of the samples investigated is given in S1 Table.

The non-synonymous *MTAP* SNP rs7023954 (c.G166A, p.Val56Ile, GenBank accession numbers XM 027613 and NM 00D2451) was genotyped by Sanger sequencing of exon 3, which had been PCR amplified from reverse transcribed *MTAP* transcripts isolated from normal skin as well as primary and metastatic melanoma cell lines and tissues (Fig 1, S1 Table). In both, normal skin and primary melanoma cell lines and tissue, the distribution of genotypes did not deviate significantly from HWE. In contrast, a highly significant (P = 0.00019) deviation from HWE, due to a deficit of heterozygotes, was observed in metastatic tumor tissue and cell lines. However, limiting the analysis to metastatic melanoma tissues only, no significant deviation (P = 0.0719) from HWE could be detected. Finally, testing for a genetic association between rs7023954 and primary melanoma using a χ^2^ test, no significant association could be found regardless whether the frequency of alleles or genotypes was considered.

### Quantitation of *MTAP* rs7023954 allelic expression at the protein level

To confirm expression of the *MTAP* rs7023954 alleles at the protein level, six cell lines homozygous for either the A- (Mel Im, Mel Ho and A375) or the G-allele (WM793, WM1366 and WM293A) and a heterozygous fibroblast cell line (3F0379) were analyzed by LC-MS/MS using allele-specific stable isotope-labeled proteotypic peptides as internal standards for accurate quantitation (Table 1). We determined isoleucine at position 56 in all samples with the AA genotype and valine in all samples with the GG genotype, respectively, while the heterozygous cell line 3F0379 showed co-dominant expression of both alleles. Expression levels ranged from 20 fmol/mg protein for Mel Ho to 2800 fmol/mg protein for A375. In the other melanoma cell lines, expression differed far less, ranging from 180 to 320 fmol/mg protein. The low level of MTAP protein expression detected in Mel Ho agreed with Western blotting performed previously (Behrmann et al., 2003). In the heterozygous cell line 3F0379, alleles were expressed at 60 fmol/mg protein each.

**Table 1:** Confirmation of cDNA genotype by liquid chromatography-tandem mass spectrometry (LC-MS/MS) based protein expression analysis. Sole expression of the 56I-allele was observed in melanoma cell lines Mel Im, Mel Ho, and A375, whereas the 56V-allele was detected exclusively in WM793, WM1366, and WM293A. In the fibroblast cell line 3F0379, both alleles were co-expressed equally.

Cell lines	Detected variant
**Mel Im**	56-I
**Mel Ho**	56-I
**A375**	56-I
**WM793**	56-V
**WM1366**	56-V
**WM293A**	56-V
**3F0379** (NHDF)	56-I/V

### Enzyme kinetics of *in vitro*-translated MTAP

**Fig 2:**
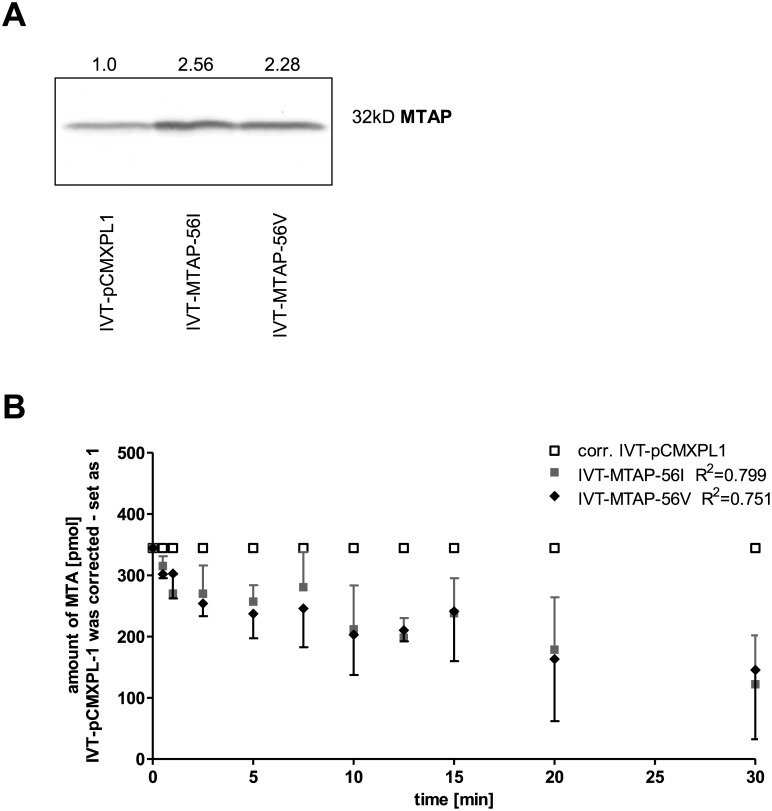
Enzyme kinetics of both *in vitro*-translated MTAP variants. (A) Western Blot for determination of MTAP expression by *in vitro* transcription/translation of the pCMX-PL1 (1.0) control plasmid and the expression constructs for MTAP-56I (2.56-fold increased) and MTAP-56V (2.28-fold increased). Densitometric quantification revealed increased MTAP amount. The original Western blot is shown in S1 Fig. (B) Analysis of the metabolic activity of the *in vitro*-translated products by liquid chromatography–tandem mass spectrometry. Catalytic rates of IVT-MTAP-56I and IVT-MTAP-56V were corrected for the rate of the control, which was set at 1.

Recombinant MTAP protein with either 56V or 56I was expressed by *in vitro* transcription/translation (IVT). The translation efficiency of pCMX-PL1 (control vector), MTAP-56V and MTAP-56I was monitored by Western Blot analysis (Fig 2A; S1 Fig). Enzyme kinetic assays showed no difference in the rate of catabolism of MTA between the alleles (Fig 2B).

### Enzyme kinetics of MTAP in transiently and stably transfected cells

**Fig 3:**
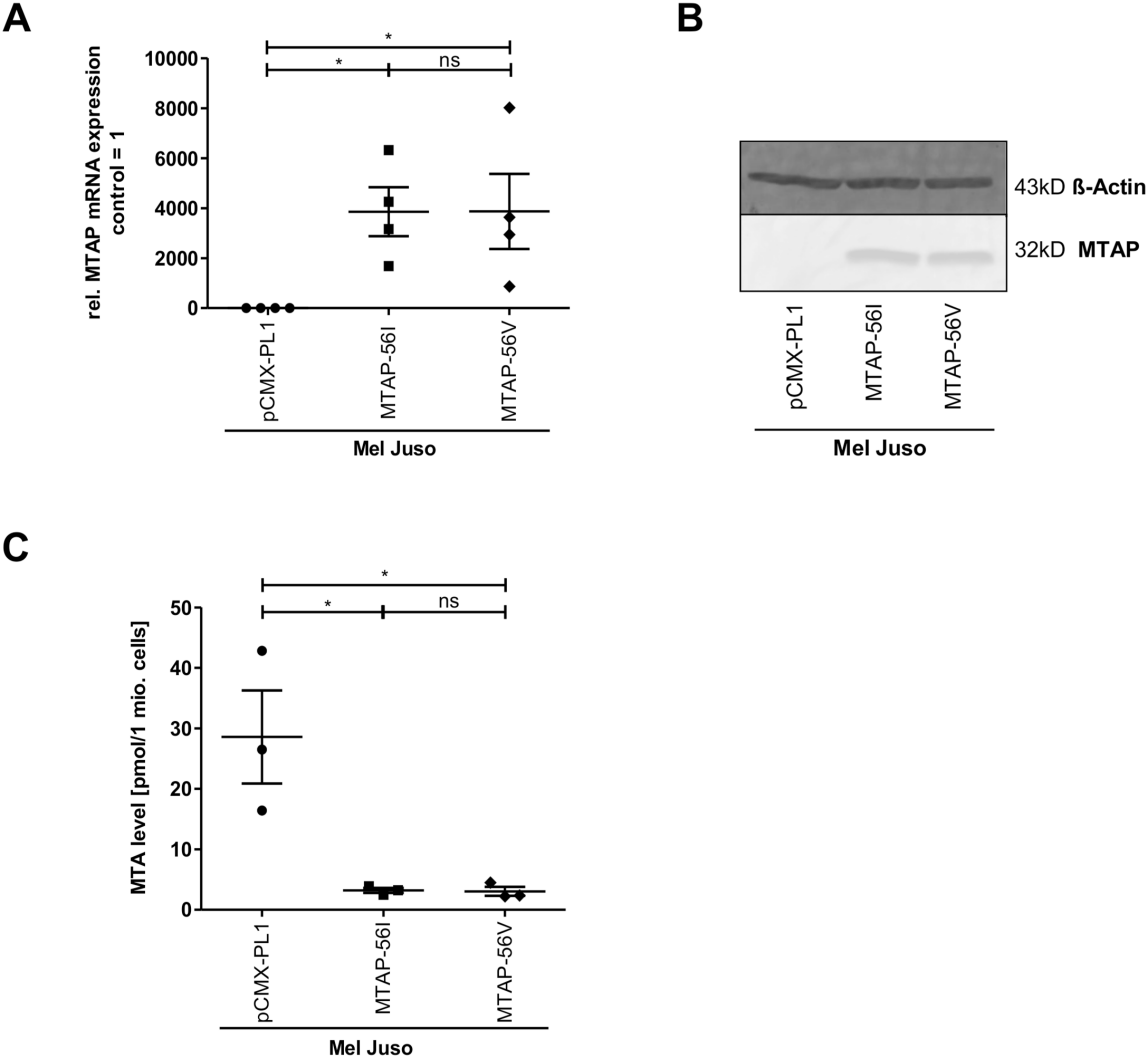
Effect of both MTAP variants in transiently cells. Quantification of MTAP expression at the mRNA (A) and protein level (B) in the melanoma cell line Mel Juso after transient transfection of expression constructs for MTAP-56I and MTAP-56V or control plasmid (pCMX-PL1). The original Western blot is shown in S2 Fig. (C) Analysis of intracellular MTA levels in the transiently transfected Mel Juso cells.

Reaction rates of the two *MTAP* alleles were also measured in cell culture. To that end, the melanoma cell line Mel Juso, which did not express any MTAP protein detectable by LC-MS/MS, was transfected with control (pCMX-PL1) vector, MTAP- 56I or MTAP-56V expression construct, respectively. Successful transfection was confirmed by qRT-PCR and Western Blot (Fig 3A). Both MTAP-56I and MTAP-56V transfected cells showed a similarly strong reduction in intracellular MTA levels compared to the control (Fig 3B; S2 Fig).

**Fig 4:**
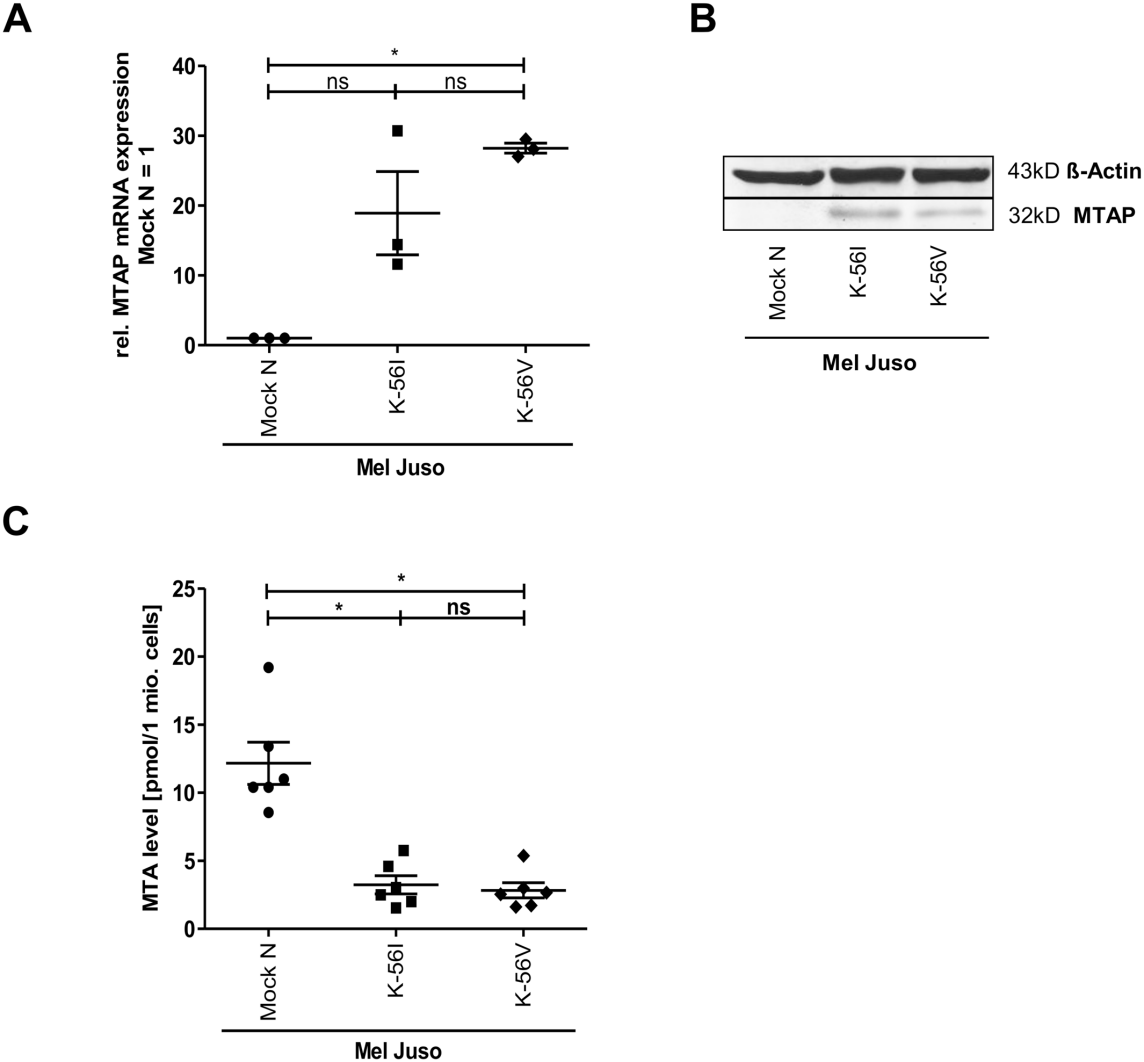
Enzyme kinetics of both MTAP variants in stably transfected cells. Quantification of MTAP expression at the mRNA (A) and protein level (B) in the melanoma cell line Mel Juso after stable transfection of control plasmid (pCMX-PL1) or expression constructs for MTAP-56I and MTAP-56V. The original Western blot is shown in S3 Fig. (C) Analysis of intracellular MTA levels in the stably transfected Mel Juso cell clones.

To quantify the differences between MTAP variants in Mel Juso after long-time re-expression of MTAP-56V and MTAP-56I, stably transfected clones were generated and analyzed in the same manner. MTAP expression was confirmed at the RNA and protein level (Fig 4A). The cells were analyzed for intracellular MTA by LC-MS/MS (Fig 4B; S3 Fig). Again, a significant reduction in MTA concentration was detected in MTAP re-expressing cell clones compared to control. The reduction in intracellular MTA concentration was again similar for the alleles.

## Discussion

The chromosomal locus 9p21 has been implicated in the pathogenesis of cutaneous malignant melanoma [26,27]. Several studies have revealed a link between 9p21 and pigmentation [27,28]. Increased pigmentation in metastatic melanoma tissue is associated with both a significant shorter overall survival (OS) and progression free survival (PFS) [29] as well as a poor response to radiotherapy [30]. The locus also harbors, aside from *MTAP*, the type I interferon (*IFN*) gene cluster, the two cyclin-dependent kinase inhibitors *CDKN2A* (encoding p16^INK4a^ and p14^ARF^) and *CDKN2B* (encoding p15^INK4b^), and a long intergenic noncoding RNA, designated antisense noncoding RNA in the INK4 locus (*ANRIL*), which has been shown to regulate *CDKN2A/B* by epigenetic mechanisms [31].

Genome-wide association studies (GWAS) have identified a number of loci predicting nevus count, including the 9p21 locus, which is also significantly associated with melanoma risk [15]. Recent studies reported a significant association of the *MTAP* SNPs rs7023329 (intron 2) and rs10811629 (intron 5) with melanoma incidence and number of melanocytic nevi [28]. Further, the *MTAP* polymorphism rs10757257*G in intron 1 was found to be significantly associated with melanoma risk in adults and, in particular, with superficial spreading and nodular melanoma subtypes [16].

To date, the non-synonymous SNP rs7023954 in exon 3 of the *MTAP* gene, which results in a conservative substitution of a branched-chain amino acid residue at position 56 for another, has not been linked to melanoma development. Here, we genotyped rs7023954 in both primary and metastatic melanoma cell lines and tissues and compared the genotype and allele counts observed to those in normal skin tissue and cell lines. We further confirmed in the fibroblast cell line 3F0379, which is heterozygous for rs7023954, the co-dominant expression of the alleles at the protein level by LC-MS/MS using allele-specific stable-isotope labeled peptides as internal standards for accurate quantitation. While the genotype frequencies observed in normal and primary melanoma samples adhered to those expected under the Hardy-Weinberg assumption, a significant deviation from HWE was found in metastatic melanoma samples due to a deficit of heterozygotes. However, considering metastatic melanoma tissue only, no significant deviation from HWE could be detected. This is not an uncommon finding in comparative high-resolution surveys of genome-wide chromosomal abnormalities in established cell lines and primary human tumors [32]. Moreover, in contrast to previous papers that had observed homozygous deletions of the *MTAP* locus in 3 out of 11 [33] and 1 out 9 investigated melanoma cell lines [6], respectively, no evidence of a diallelic deletion of the *MTAP* locus was found here. This supports our previous finding, that loss or reduction of MTAP expression in melanoma is more often the result of hypermethylation of the promoter region than chromosomal deletion of the *MTAP* locus [6].

Tests of genetic association performed on observed allele counts in normal and primary melanoma samples showed a positive, albeit not significant association of *MTAP* rs7023954*G (OR 1.746, 95% CI = 0.6928–4.4006) with melanoma. It cannot be excluded that the lack of significance is a consequence of the small sample size studied and the resulting lack of statistical power. As a matter of fact, the odds ratio of 1.746 observed for *MTAP* rs7023954*G is quite similar to that reported for *MTAP* rs10757257*G and melanoma risk in adults (OR = 1.32, 95% CI = 1.14–1.54). This led us to investigate whether the G allele might differ in enzymatic activity from the A allele. We cloned both alleles and expressed them using both a rabbit reticulocyte lysate translation system as well as transient and stable transfection of an *MTAP*-null melanoma cell line. In all three systems, enzyme kinetics of the alleles did not differ significantly, indicating that a difference in catabolism of MTA is unlikely to contribute to melanoma development and progression. This concords with a recently published study that found a catalytically inactive version of *MTAP*, which was introduced into HT1080 fibrosarcoma cells, to be fully capable of reversing various tumor phenotypes such as soft agar colony formation and increased migration and metalloproteinase production [34].

## Conclusion

The absence of a difference in the rate of catabolism of MTA to adenine and 5-methylthioribose-1-phosphate between the alleles of the only common amino acid substitution polymorphism described to date for *MTAP* lends further support to the notion, that the tumor suppressive function of *MTAP* is independent of its known enzymatic activity.

## Supporting Information

S1 Fig Original Western blot of Fig 2A. Two independent Western blots for the determination of MTAP expression by *in vitro* transcription/translation.
(EPS)

S2 Fig Original Western blot of Fig 3B. Two independent Western blots for the determination of MTAP expression after transient transfection.
(EPS)

S3 Fig Original Western blot of Fig 4B. Two independent Western blots for the determination of MTAP expression after stable transfection.
(EPS)

S1 Table Genotyping of MTAP rs7023954. Distribution of the genotypes AA, AG, and GG of SNP rs7023954 determined by cDNA amplicon sequencing in various normal skin as well as primary (prim.) and metastatic (met.) melanoma cell lines and tissues.
(DOCX)
