# Supplementary material for: Characterization of the Methylthioadenosine Phosphorylase Polymorphism rs7023954 - Incidence and Effects on Enzymatic Function in Malignant Melanoma
Source: PLoS One. 2016 Aug 1;11(8):e0160348. doi: 10.1371/journal.pone.0160348 (PMC4968798; doi:10.1371/journal.pone.0160348)
Supplement: S1 Table — Distribution of the genotypes AA, AG, and GG of SNP rs7023954 determined by cDNA amplicon sequencing in various normal skin as well as primary (prim.) and metastatic (met.) melanoma cell lines and tissues. (DOCX) [file pone.0160348.s004.docx]

**S1 Table.**

| **Cell lines and tissue samples** | **c.166A** | **c.166AG** | **c.166G** |
| --- | --- | --- | --- |
| **NHEM, NHDF and normal tissue** | NHEM1  NHEM2  NHDF1  NHDF2  TB88  TB89  TB122  NH120 | NHEM3  NHDF3  NHDF4  NHDF5  NHDF6  NH119 | NHEM4  NL3  NH14  NH118  TB121  NL146 |
| **Primary melanoma cell lines and tissue** | Mel Ei  Mel Wei  Mel Ho  WM3211 | WM35  TB71  TB87  TB93  TB97  TB148 | Mel Juso  SbCl2  WM793  WM1366  TB60  TB62  Tb104 |
| **Metastatic melanoma cell lines and tissue** | 501Mel  HMB2  A375  Mel Im  TB135 | TB90 | SkMel3  SkMel28  Mel Ju  WM9  WM293A  TB43  TB50  TB80  TB91  TB95  TB101  TB147 |
